# Elevated Atmospheric Co_2_ Levels Impact Soil Protist Functional Core Community Compositions

**DOI:** 10.1007/s00284-024-03930-3

**Published:** 2024-10-16

**Authors:** Alessandra Ö. C.-Dupont, David Rosado-Porto, Indhu Shanmuga Sundaram, Stefan Ratering, Sylvia Schnell

**Affiliations:** 1 Institute of Applied Microbiology, IFZ, Justus-Liebig-University Giessen, Heinrich-Buff-Ring 26-32, 35392 Giessen, Germany; 2 Present Address: Ceradis Crop Protection BV, Agrobusiness Park 10, 6708 PW Wageningen, Netherlands

## Abstract

Protists, known as microeukaryotes, are a significant portion of soil microbial communities. They are crucial predators of bacteria and depend on bacterial community dynamics for the growth and evolution of protistan communities. In parallel, increased levels of atmospheric CO_2_ significantly impact bacterial metabolic activity in rhizosphere soils. In this study, we investigated the effect of elevated atmospheric CO_2_ levels on the metabolically active protist community composition and function and their co-occurrences with bacteria from bulk and rhizosphere soils from the Giessen Free-Air CO_2_ enrichment grassland experiment. Metabarcoding sequencing data analyses of partial 18S rRNA from total soil RNA showed that elevated CO_2_ concentrations stimulated only a few ASVs of phagotrophic predators of bacteria and other microeukaryotes, affecting protist community composition (*P* = 0.006, PERMANOVA). In parallel, phagotrophic and parasitic lineages appeared slightly favoured under ambient CO_2_ conditions, results that were corroborated by microbial signature analyses. Cross-comparisons of protist-bacteria co-occurrences showed mostly negative relations between prokaryotes and microeukaryotes, indicating that the ongoing increase in atmospheric CO_2_ will lead to changes in microbial soil communities and their interactions, potentially cascading to higher trophic levels in soil systems.

**Supplementary Information:**

The online version contains supplementary material available at 10.1007/s00284-024-03930-3.

## Introduction

The steady rise in atmospheric carbon dioxide (CO_2_) concentration due to human activities has brought up concerns regarding its impact on the Earth's ecosystems, with changes occurring at very high levels for most ecosystems regarding not only structure but also species shifts (IPCC 2022). Terrestrial ecosystems, particularly soils, are vital in regulating global carbon cycling. Soils represent some of the most biodiverse ecosystems on Earth, harbouring organisms ranging from the micro- to the macro-scale. Soil microorganisms—bacteria, archaea, protists and fungi—are particularly abundant and significant drivers of nutrient cycles, pathogen control and suppression. Unicellular microeukaryotes, or protists, a taxonomically, morphologically, and ecologically diverse group of microorganisms ubiquitous in soil environments, are key players in environmental processes such as nutrient cycling and ecological interactions. Primary predators of bacteria, phagotrophic protists directly modulate bacterial biomass and community composition, thus promoting eco-evolutionary dynamics in soil microbial populations as well as nutrient cycling [1–4].

While much research has focused on the responses of plants and bacteria to elevated CO_2_ levels, the role of protists in soil ecosystems under these conditions has received less attention [5–7]. Elevated CO_2_ levels can affect soil properties and processes, including nutrient availability, microbial activity, and organic matter. This leads to indirect changes in soil microbial communities, potentially impacting nutrient cycling and ecosystem functioning [8]. Elevated CO_2_ can alter plant root exudation patterns, influencing soil microbial activities and carbon turnover rates [9]. The increase in temperature associated with rising CO_2_ concentrations can also directly affect the growth rates and physiology of protists, in particular population dynamics such as feeding behaviour, competitive interactions, etc., as well as abundance, diversity and community composition [10]. Indirect effects can arise from shifts in plant species distribution [11], physiology, root exudation patterns, organic matter and nutrient availability [12], which can impact protist-mediated processes in soils. However, the responses of protists to abiotic conditions, such as CO_2_ levels, are highly complex and context-dependent, often varying with different soil types, plant species, and other environmental factors [13]. This is why it is necessary to tackle specific drivers to create knowledge which can be used for future research.

The Giessen Free-Air CO_2_ Enrichment (Gi-FACE) experiment in Hesse, Germany, is one of the longest-running experiments of this type in Europe. It submits typical grassland ecosystems to either ambient-air (aCO_2_) or air with + 20% CO_2_ concentrations (eCO_2_) to observe long-term changes linked to atmospheric CO_2_ concentrations.

Different studies have analysed the impact of CO_2_ enrichment on the Gi-FACE grassland ecosystems, with contrasting results [14–16]. Regardless of contradictions, Rosado-Porto and colleagues determined that elevated air CO_2_ concentrations significantly altered carbon and nitrogen cycle dynamics in the Gi-FACE rhizosphere soils by modulating bacterial community composition [17, 18].

The microbiome implies the bacterial, and eventually, archaeal, component of microorganisms associated with a host or specific environment, while the mycobiome is now commonly used to address its fungal component. For this study, we addressed the soil consumer “eukaryome”, or the protist component of soil-associated microorganisms that prey on bacteria. The biome composition between environments or hosts can be very different, but the same groups of microorganisms are often found in different biomes due, for example, to similar ecology. Groups of similar microorganisms found in different biomes are called "core" (micro)biomes. The “core microbiome” initially defines the group of microbial taxa common to all or most humans [19, 20], and the definition has been extended to include non-human hosts and systems [21]. In our environmental study context, we use the term “core eukaryome” to define the metabolically active protists that occur at higher levels in spatially distributed samples in varied environmental conditions. While taxonomic analyses of the metabolically active (core) eukaryome—that is, metabarcoding analysis of 18S rRNA rather than 18S rRNA genes—inform on which microorganisms are present where and at which abundance and proportions, they do not account for trophic function [22]. Although function is mostly redundant in natural environments, coupling it with taxonomic affiliation allows us to determine which microorganisms drive ecological changes in the considered system. Indeed, drastically different microorganisms might carry the same function, occupying different niches [23] and driving similar or opposite biotic and abiotic changes, or both.

While bacterial and, to a certain extent, fungal responses to elevated atmospheric CO_2_ have been reasonably well studied in soil systems [24–26], the protists’ reaction has only been partially investigated in terrestrial or soil systems [5, 6]. In this study, we analysed the response of consumer protists to changes in CO_2_ air conditions, and rather than looking solely at the taxonomic composition of the metabolically active core eukaryome, we also analysed their functional distribution.

## Material and Methods

### Sample Collection

Soil samples were collected in September 2017 at the Giessen Free-Air CO_2_ Enrichment (FACE) station located at the “Environmental Monitoring and Climate Research Station Linden”, jointly maintained by the Justus-Liebig-University and the Hessian State Agency for Nature Conservation, Environment and Geology. The facility consists of 6 rings with an 8 m diameter, three rings representing an ambient-air treatment (no CO_2_ enrichment) and the other three rings of an elevated CO_2_ concentration treatment. Elevated CO_2_ rings have been enriched with 20% above-ambient CO_2_ concentrations during daylight hours since May 1998. The area was managed as a meadow before the facility’s establishment, and the vegetation is dominated by 12 species, with *Arrhenatheretum elatioris* Br.Bl. and *Filipendula ulmaria* as major components. Since 1996, the grassland has been fertilised with 40 kg ha^−1^ a^−1^ nitrogen [27].

Core soil samples were collected with cut 50 mL syringes (11 × 3 cm) to a depth of 10 cm and treated as described in [15]. Three replicates from each ring were collected, and samples were stored in ice until they were processed in the laboratory. Soil samples were separated into rhizosphere soil that adhered to plant roots and bulk soil, processed through a 2 mm sieve to homogenise samples, and frozen at − 80 °C. Samples’ processing occurred in less than 2 h after sampling.

### Nucleic Acid Extraction and Sequencing

RNA extraction was performed according to a modified protocol from Mettel et al. [28], described in detail in [17]. Shortly, cells were disrupted in a cell mill MM200 (Retsch, Germany) and precipitated; the supernatant was collected and treated for a DNA/RNA phenol/chloroform extraction. The resulting DNA/RNA pellet was dissolved in 50 µL of nuclease-free water. Samples were then treated with RNase-Free DNase Set (QIAGEN GmbH, Germany) for DNA digestion according to the manufacturer’s instructions. The DNase reaction was stopped with 10 µL of 50 mM EDTA, and the samples were checked for DNA absence by PCR with universal primers. Reverse transcription was performed following the manufacturer protocol of the AccuScript High Fidelity 1st Strand cDNA Synthesis Kit (Agilent Technologies, Inc., Cedar Creek – Texas, USA).

A partial sequence of the V4 region of the eukaryotic 18S small subunit ribosomal RNA (SSU rRNA) of the newly synthesised cDNA was amplified with the primers V4_1f (CCAGCASCYGCGGTAATWCC) and TAReukREV3 (ACTTTCGTTCTTGATYRA) according to the protocol in [29]. The bacterial 16S rRNA hypervariable regions (V4&V5) were sequenced after PCR amplification of the transcripted cDNA using the primers 520F (5’-AYTGGGYDTAAAGNG-3’) [30] and 907R (5’-CCGTCAATTCMTTTRAGTTT-3’) [31]. PCR products were purified and sequenced with an IonTorrent sequencer (Thermo Fischer Scientific, PGM) as described in [32].

Microeukaryotes sequencing data were imported into QIIME2 version 2021.8 [33] for quality filtering and preliminary data analysis. Sequences were demultiplexed with cutadapt [34], with 0 barcode error rates and sorted according to samples’ barcodes. Demultiplexed sequences were quality filtered, denoised, dereplicated, and chimaeras filtered out with the implemented DADA2 [35] plugin in QIIME2; sequences were trimmed at nucleotide position 15 as recommended by the authors of DADA2 and truncated at 300 nucleotides length, and grouped in representative sets of amplicon sequence variants (ASVs). Taxonomic affiliations were obtained via a trained fitted Naive Bayes classifier [36] based on the Protist Ribosomal database PR2 4.14.0 [37]. ASVs with three reads or less in only one sample were removed, and ASVs present in at least 80% of all samples were kept as “core ASVs”. Core eukaryomes were individually calculated for all soil and CO_2_ conditions (rhizosphere ambient, rhizosphere elevated, bulk ambient and bulk elevated) with the core function from the package microbiome [38], resulting in 56 ASVs over 12 samples.

Bacterial sequences were first demultiplexed with the QIIME2 cutadapt command, using a barcode error rate of zero and assigned to specific samples by corresponding their barcodes. Later, quality control, denoising, sequence dereplication, and chimaera filtering were performed using the DADA2 plugin in QIIME2. The first 15 nucleotides were trimmed as recommended by the authors of DADA2, and sequences were truncated at 320 nucleotides. Amplicon sequence variants generated with DADA2 were taxonomically affiliated with a trained fitted classifier based on the SILVA 138.1 database [39, 40]. Finally, ASVs classified as chloroplast and mitochondria were removed from the frequency table and representative sequences.

This study focused on heterotrophic protists and their eventual interactions with bacteria and their environment. ASVs corresponding to plants (Embryophyceae, 17 ASVs), animals (Metazoa, 331 ASVs), true fungi (Dicarya or Fungi, 828 ASVs), and other strictly photosynthetic organisms (Chlorophyta and non-phagotrophic Ochrophyta, 121 ASVs) were filtered out before further analyses. Likewise, the analyses did not include sequences identified as “Eukaryota” only (263 ASVs).

### Trophic Functional Assignment

Functional traits classification was curated manually by compiling information in [41], the Protist Interaction Database (PIDA, [42]) and the Cercozoa-Endomyxa functional traits table available on GitHub.com (https://github.com/Kenneth-Dumack/Functional-Traits-Cercozoa-Endomyxa#functional-traits-cercozoa-endomyxa). Microeukaryotes were classified according to their trophic characteristics, as saprotrophs, lysotrophs, osmotrophs, detritivores and consumers. Saprotrophs or lysotrophs are involved in extracellular enzymatic digestion, typically when degrading organic matter and osmotrophs have a diet exclusively based on nutrient obtention by passive diffusion of soluble substrates between the environment and their intracellular compartment (strict osmotrophy). Detritivores ingest particles found in the environment that do not contain living cells. Consumers are microeukaryotes that eat other species, either by pursuing prey and ingesting it, such as predators, or grazers that do not actively pursue prey but rather collect them from an available nearby pool. Consumers may ingest prey (bacteria) either by filtration or by phagocytosis. Cytotrophy identifies protists known to ingest bacteria and other eukaryotes, particularly other protists. Those whose diet is composed explicitly of microalgae were further characterised as phycotrophs, while those ingesting fungi only were classified as mycotrophs. Intracellular consumers of animals, plants or other microeukaryotes were categorised as parasites. Finally, protists that are either primarily cytotrophs that keep prey plastids for photosynthesis or phototrophs that occasionally ingest food by phagocytosis were categorised as mixotrophs (mixotrophy). In the case of Ciliophora, which usually ingest bacteria by filtration, specific terms identify omnivores (ciliates that occasionally ingest small protists in addition to bacteria) and predators (consumption not by filtration).

### Statistical analyses

All statistical, diversity and differential abundance analyses were done with R studio software version 2021.09.1 + 372 (R core Team 2021) with packages Phyloseq 1.40.0 [43], vegan 2.6–4 [44], ALDEx2 1.26.0 [45], coda4microbiome 0.1.4 [46], and their respective dependencies.

In detail, alpha-diversity differences between soil habitats and CO_2_ conditions were calculated with the Wilcoxon test [47]. Beta-diversity was analysed by centred-log-ratio transformation of the core eukaryome data followed by calculation of Aitchison distances [48] and visualised with principal components analysis in R. Differential abundance analysis between ambient and elevated atmospheric CO_2_ conditions with ALDEx2 was performed by creating 128 Monte Carlo instances for each sample’s features and then performing a centred log-ratio (clr) transformation of these instances. Features were considered significantly differentially abundant when showing an effect size >|0.5|.

Coda4microbiome signatures were obtained with the function coda_glmnet, which performs a penalised logistic regression on pairwise log ratios between the mean abundances for each taxa pair of the core eukaryome dataset. The penalisation parameter lambda used for the model was chosen as the minimum mean cross-validated error value (lambda = “lambda.min”), with ten folds.

Trophic functional differences according to CO_2_ conditions were tested with the adonis2 function (which performs a PERMANOVA) from the vegan package, with 999 permutations.

Co-occurrence analysis was performed using the protists and bacteria ASVs from ambient CO_2_ and elevated CO_2_ rings, which showed an ALDEx2 effect size > 0.4 [49]. Later, ASVs were analysed with the R package Spiec-easi 1.1.1 [50], applying the neighbourhood selection method [51], a lambda path number of 100, a lambda minimum ratio of 0.1 and the Stability Approach to Regularization Selection (StARS) using its default settings. Subsequently, the network visualisation was performed on Cytoscape 3.8.2 [52]. Comparisons of network topological features, such as network degree, betweenness centrality, clustering coefficients and eigenvector centrality between protist and bacteria under elevated or ambient CO_2_ conditions, were done with the Wilcox.test in R. Values were obtained with the Network Analyzer from Cytoscape, and the plugin CentiScape [53].

Correlations between ASVs and environmental parameters were calculated with R packages phyloseq, vegan, ALDEx2 and tidyverse, then visualised with ggplot2. Correlation tests were done with the function Aldex.corr with the Kendall correlation coefficient. *P*-values were corrected with the false discovery rate (FDR) method with alpha < 0.05.

## Results

### Ion Torrent Sequencing

Raw eukaryotic sequences (390,309 input sequences) were demultiplexed into 12 samples corresponding to the six Giessen FACE facility rings, three of which at ambient-air CO_2_ levels (aCO_2_, rings A1, A2 and A3) and three with elevated CO_2_ levels (eCO_2_, rings E1, E2 and E3). After quality filtering, denoising, and dereplications, 173,053 non-chimeric sequences remained and were grouped into 2534 ASVs.

The removal of ASVs corresponding to non-phagotrophic protists and other eukaryotes (see Trophic functional assignment in Methods) and ASVs identified no further than “Eukaryota” resulted in a final set of 974 ASVs. The final core eukaryome used for all analyses comprised 56 ASVs (SI Table 1).

From 560,371 bacterial input sequences, 358,161 non-chimeric sequences were kept after quality filtering, denoising, dereplication and removal of chloroplast and mitochondria, and finally grouped into 4019 ASVs.

Due to the facility’s limitation in sampling quantities, the sequencing effort was sufficient for all samples despite the small number of samples. Rarefaction curves show a plateau for all samples, even if at a low number of sequences.

### Taxonomic Community Composition at CO_2_ Levels in Bulk and Rhizosphere Soils

**Fig. 1:**
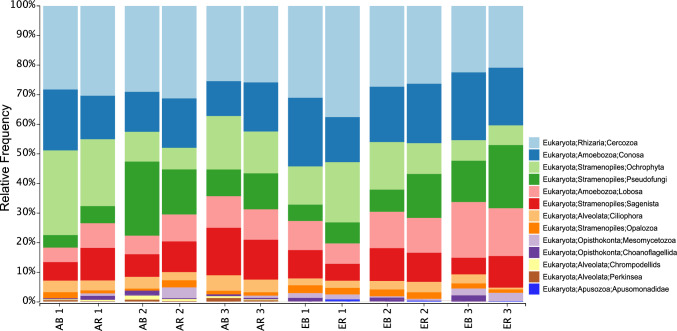
Soil microbial taxonomic diversity under ambient (bars AB1 to AR3) and elevated (bars EB1 to ER3) CO_2_ conditions collapsed at the division (L3) level. Numbers 1–3 indicate the Gi-FACE facility rings

In all four conditions, active protist communities were dominated by Stramenopiles, Rhizaria and Amoebozoa (Fig. 1). In addition to those phyla, rhizosphere soils from elevated CO_2_ conditions also contained ASVs belonging to the Apusozoa.

**Fig. 2:**
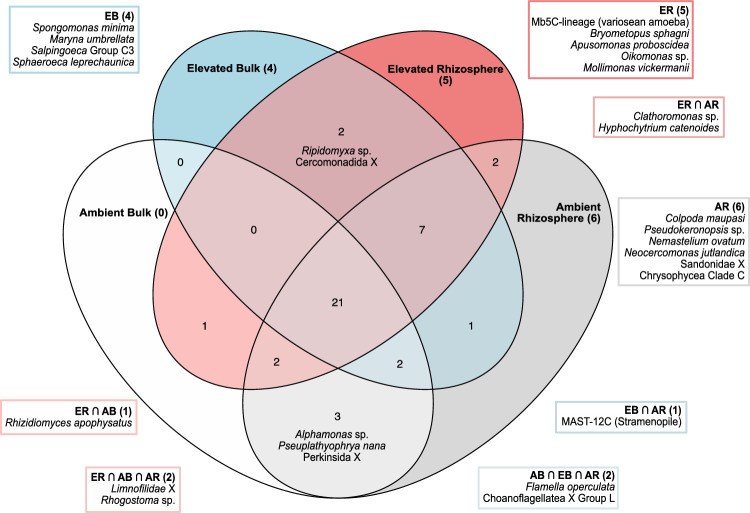
Distribution of the ASVs belonging to the 80%-occurrence core “eukaryome” in bulk and rhizosphere soils under ambient or elevated CO_2_ conditions. *AR* ambient rhizosphere, *ER* elevated rhizosphere, *AB* ambient bulk, *EB* elevated bulk, (intersection): indicate overlapping conditions as illustrated in the Venn diagram. Numbers in the corresponding boxes indicate the total ASVs found for each specific condition (AR, ER, AB, etc.)

Of all 56 ASVs composing the selected core eukaryome, 21 were shared by all conditions. At the same time, five were exclusively found in rhizosphere soils in elevated CO_2_ conditions, four in elevated CO_2_ bulk soils, and six in rhizosphere soils under ambient CO_2_ conditions. No ASVs were exclusively found in bulk soils under ambient CO_2_ conditions (Fig. 2).

### Alpha- and Beta-Diversity Analyses

Differences in protist communities between soil habitat and CO_2_ concentrations were assessed with species richness (observed) and alpha-diversity indices Shannon (SI Fig. 1a), Simpson and Fisher (data not shown). Species richness was significantly higher in rhizosphere soils when compared to bulk soils in both CO_2_ conditions (*P* = 0.002). The same was observed for bulk soils at high and ambient CO_2_ levels (aBulk < eBulk, *P* = 0.04). However, rhizosphere soils decreased in richness under elevated CO_2_ conditions (aRhizo > eRhizo, *P* = 0.004). No significant differences were observed for Shannon (SI Fig. 1a), Simpson and Fisher indices (data not shown) for all soil and CO_2_ level comparisons.

Significant differences between samples according to CO_2_ level were corroborated with beta-diversity analysis (PERMANOVA, *P* = 0.001; SI Fig. 1b), and samples were also significantly different according to soil habitat in this analysis (bulk vs. rhizosphere, PERMANOVA *P* = 0.027).

### Differential Abundance Analyses

Differential abundance analyses indicated only a few differences between soil and CO_2_ conditions. ALDEx2 identified four differentially abundant taxa when comparing bulk to rhizosphere soils: *Flamella operculata* and *Euglypha rotunda* were enriched in bulk soils, while *Hyphochytrium catenoides* and *Clathromonas butcheri* appeared enriched in rhizosphere soils (SI Fig. 2a). In parallel, in ambient versus elevated CO_2_ soils comparisons, *Pseudoplatyophrya nana*, *Alphamonas* sp., and *Perkinsida* sp. were enriched in ambient CO_2_ soils while two unidentified Variosean (Variosea sp. and WIM-1-lineage sp.), an unidentified Cercomonadidae and a *Ripidomyxa* sp. were enriched in elevated CO_2_ soils (SI Fig. 2b). More specifically, differential abundance in individual treatments was analysed in ambient against elevated CO_2_ conditions in either rhizosphere or bulk soils or in bulk soils compared to rhizosphere soils in either elevated or ambient CO_2_ conditions. Significant differences in abundance were only found when comparing rhizosphere against bulk soils in ambient CO_2_ conditions (7 enriched taxa in aCO_2_ bulk soils).

**Fig. 3:**
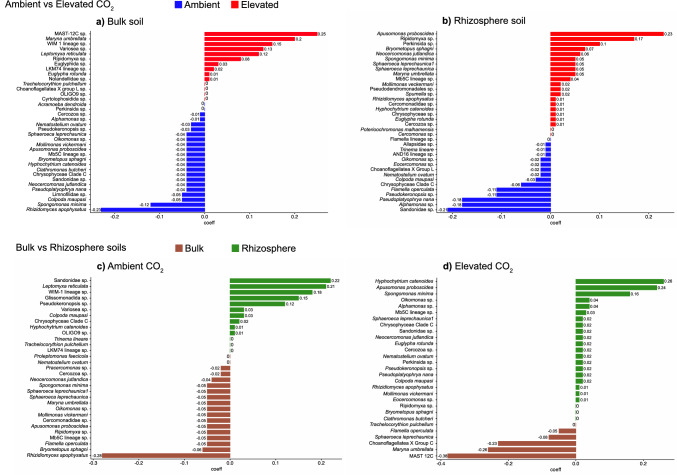
Microbial signatures of **a** bulk and **b** rhizosphere soils according to atmospheric CO_2_ conditions and signatures for soils under **c** ambient and **d** elevated CO_2_ conditions. Groups with positive regression coefficients contribute more to either the composition of communities under elevated CO_2_ conditions (red bars, a and b) or the characterisation of rhizosphere soil communities (green bars) under ambient (c) or elevated (d) CO_2_ conditions

For microbial signatures (Fig. 3a-d), ASVs with the highest regression coefficients (> 0.1) are primarily characteristic of their environment. While most signatures identified the same groups in the same conditions, it is interesting to notice that *Spongomonas minima* was present in both "bulk ambient” (Fig. 3a) and “ambient rhizo” (Fig. 3c) soils. Likewise, the WIM-1 lineage ASV and *Leptomyxa reticulata* were found in both “bulk elevated” (Fig. 3a) and “ambient rhizo” (Fig. 3c) soils. Permutational tests showed that the mean cv-AUC value for the tested conditions (soil habitat and CO_2_ level) is significantly different from those expected under the null hypothesis, thus confirming that observed microbial signatures are characteristic of soil habitat and CO_2_ conditions and not due to chance.

### Functional Community Composition and Network Analysis

**Fig. 4:**
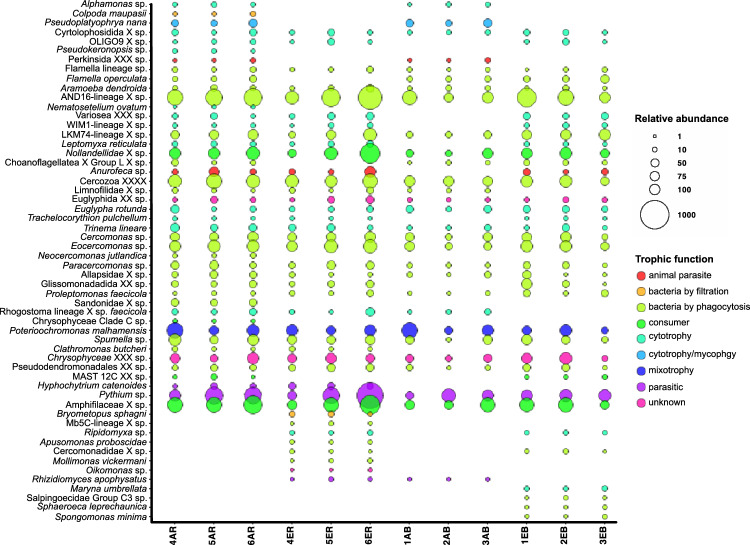
Functional bubble plot of the 80%-occurrence core eukaryome of rhizosphere (R) and bulk (B) soils under ambient (A) and elevated (E) CO_2_ conditions of the different rings (1–3). Bubble colours correspond to the protists’ trophic functions, while bubble sizes indicate the ASVs’ abundance

To better characterise differences observed between protist communities at elevated and ambient CO_2_ levels, protists were classified according to their functional traits, more precisely according to their trophic roles (Fig. 4).

Analysis of ASVs identified as microbial signatures for CO_2_ conditions and soil types indicated that core communities are dominated by cytotrophs, with only *Flamella operculata* being a specific bacterivore and *Perkinsida* sp. and *Hyphochytrium catenoides* parasites.

**Fig. 5:**
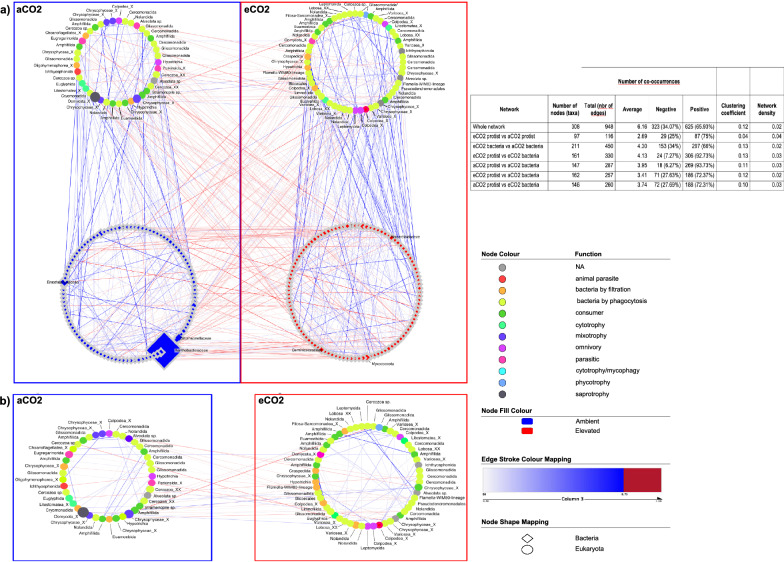
**a** Protist-bacteria co-occurrence network (upper rings – protists, bottom rings – bacteria) and **b** protist-protist co-occurrences in soils under ambient (blue frames, left) and elevated (red frames, right) CO_2_ conditions for combined soil types (rhizosphere and bulk). Only ASVs with an ALDEx2 effect size greater than 0.3 (protist) and 0.4 (bacteria) are shown in the networks. Bacterial ASVs are represented by diamonds and protists in circles. Protistan nodes are coloured according to their functional traits (legend). Edges are coloured blue for positive correlations and red for negative correlations

Network analysis identified which protists and bacteria potentially co-occurred in ambient and elevated CO_2_ conditions. Globally, microorganisms under similar CO_2_ conditions showed mostly positive co-occurrences. In contrast, co-occurrences between microorganisms between elevated and ambient CO_2_ conditions were mainly negative (Fig. 5a), indicating possible specific co-occurrences linked to environmental CO_2_ conditions. However, protist-bacteria correlations within CO_2_ conditions indicated much higher positive co-occurrences (92.73% and 93.73% for elevated CO_2_ and ambient CO_2_ respectively) than negative ones (7.27% and 6.27%) (Fig. 5). However, no significant differences were identified in network centrality indices – degree, clustering coefficient, betweenness and eigenvector – between protist and bacteria (SI Fig. 4) and CO_2_ conditions (SI Fig. 5). Interestingly, in ambient CO_2_ conditions, one *Bradyrhizobium* sp. (*Xanthobacteraceae*, Fig. 5a) ASV is very abundant. However, it shares edges with only nine other ASVs, of which one protist ASV from elevated CO_2_ conditions, a *Nolandellidae* sp.

To identify stronger correlations between microorganisms, hub nodes with the highest degree levels (10–23) were isolated, thus providing a sub-network of the most connected nodes in the main network (SI Fig. 3). This subnetwork comprised 37 nodes, of which 12 corresponded to microeukaryotes, 17 to prokaryotes, and 53 edges. Interestingly, most nodes belonged to elevated CO_2_ levels (25 nodes, of which 16 bacteria and nine protists), and were connected by most edges in this sub-network. Hub-protists in ambient CO_2_ conditions did not show any edges between themselves or co-occurrence to hub-bacteria in ambient CO_2_ conditions, and only a few, mostly negative, co-occurrences with either microorganism kingdoms in elevated CO_2_ conditions.

Protist-protist co-occurrences in elevated and ambient CO_2_ levels were analysed independently. A low significant difference (Wilcoxon, *P* = 0.013) was found in betweenness centrality, with the protist network under elevated CO_2_ conditions showing a slightly lower value for this index than the network under ambient CO_2_ conditions (SI Fig. 5a). Most co-occurrences were primarily positive and often involved predators and their prey, like the strong positive correlation between variosean amoebas and Allapsidae sp. or putative parasite-host or symbiotic interactions. Similarly, most protist-bacteria co-occurrences indicated possible predator–prey interactions or symbiotic interactions (Fig. 5b), although no significant differences in co-occurrences were detected (PERMANOVA).

## Discussion

Human activities have significantly impacted the Earth's environment and ecosystems in the last century. In particular, elevated CO_2_ concentrations correlate to increased microbial metabolic activity in rhizosphere soils [17]. Protists, some of the most abundant predators of bacteria in soils, directly depend on bacterial prey and indirectly depend on factors impacting bacterial communities. In this study, analyses were done with a particular focus on protists that are most likely to interact with bacteria and other protists directly. Specifically, microeukaryotes categorised as “consumers” in trophic systems. For that reason, all ASVs assigned to autotrophic and saprotrophic microorganisms were not included, although fungi and many photosynthetic microeukaryotes also closely interact with phagotrophic protists [54, 55].

The core protist eukaryome was obtained by extracting and merging 80%-occurrence cores for the four different soil conditions (ambient bulk, elevated bulk, ambient rhizosphere and elevated rhizosphere) after data trimming, resulting in a small dataset of 56 ASVs distributed over 12 samples. How to calculate the core eukaryome, or even whether to analyse eukaryome data by selecting a defined sub-dataset rather than the full dataset, can be controversial, particularly when defining the cut-off used for the core selection [56]. The method used here was chosen to maximise the intrinsic characteristics of each soil condition while minimising information loss that would have been caused by selecting a core eukaryome of the whole combined dataset. Indeed, the core protist eukaryome obtained without previous separation of environmental conditions selected for 24 ASVs for a 75% co-occurrence threshold and 8 ASVs for a core eukaryome with 80% co-occurrence, much too small datasets to be able to draw any robust conclusions.

Alpha diversity indices indicated no statistical differences in samples’ richness according to soil type and atmospheric CO_2_ concentrations, most likely due to our reduced sample size. We had expected significantly lower diversity indices in bulk soils since plant exudation in the rhizosphere creates nutrient-rich environments, optimal for bacteria and their protist predators [57]. The observed reduced species richness in rhizosphere soils under elevated CO_2_ conditions is likely because enriched environments specifically select for highly productive (opportunistic) protist lineages, which easily outcompete those with elevated ecological fitness costs [58], resulting in a low-diversity community [59]. The opposite is also possible, where the rhizosphere counter-selects for microorganisms under elevated CO_2_ conditions, for example, via plant (antimicrobial) exudates, which can directly or indirectly lead to less diverse protist communities [60]. This mechanism is most likely not observed in bulk soils where the influence of the rhizosphere is minimal, and microorganisms are not particularly advantaged according to their ecological strategies in this environment or affected by plant exudates.

Protist community composition differed according to CO_2_ level, in line with previous findings of changing protist abundances under different CO_2_ conditions [6, 7, 61]. Differential abundance analyses and microbial signatures showed that specific protist lineages were responsible for characterising communities under elevated and ambient CO_2_ conditions. Phagotrophic predators of bacteria, but also cytotrophs that can ingest larger prey, such as the variosean amoebas, were more abundant under elevated CO_2_ conditions, while parasitic (Perkinsida X) and cytotrophic lineages were partly favoured under ambient CO_2_ conditions. Interestingly, [67] observed no particular effect of CO_2_ on protist diversity or functional composition in rice paddy soils but identified O_3_ as a major driver of eventual changes. Protist interactions, however, were shown to decrease under elevated CO_2_ conditions. Similarly, [6] uncovered that elevated CO_2_ effects on microbial communities, such as the increase in bacterivorous amoeba, were strongly correlated to N fertilisation and plant cultivars. In this study, the composition of core protist eukaryomes (beta-diversity) did not significantly correlate with any other environmental parameter (C%, N%, NH_4_^+^, etc., available from [17], but for CO_2_ air concentration (SI Fig. 7).

The functional characterisation of protist communities did not change significantly according to CO_2_ concentration. Communities were largely dominated by consumers of bacteria by phagocytosis, followed by cytotrophic predators able to ingest bacteria and other micro- and small eukaryotes, unsurprisingly. Parasitic lineages, although in the minority, were more abundant either under normal atmospheric CO_2_ concentrations and/or in rhizosphere soils, suggesting links between host availability and indirect effects of aboveground environmental conditions.

Protist-bacteria correlations were mainly positive within the same atmospheric conditions but mostly negative between elevated and ambient CO_2_. It must be considered here that ASVs from different samples are gathered in one analysis and that links between nodes in our networks indicate statistical correlations but not (necessarily) physical interactions. While actual interactions can’t be confirmed in this context, it is possible to say that atmospheric CO_2_ concentration is a common selector of network components, and that network complexity slightly increases with elevated CO_2_. Interestingly, under elevated CO_2_ conditions, alpha-diversity was also lower in rhizosphere soils than in ambient CO_2_ conditions. Li and colleagues [62] similarly showed that network complexity increased with bacterial community dilution, or communities assumed to be more different regarding species composition to the original inoculum, although no assumptions could be made that those communities had more specific ecological interactions. However, *Pseudomonas*, *Burkholderia* or *Bacillus* for example, some among the many bacterial genera enriched in elevated CO_2_ soils [17], are known prey of choanoflagellates or amoebozoans [63], and some *Pseudomonas* genera can establish symbiotic (beneficial or detrimental) relationships with some amoebozoans [64].

Network analyses showed protist-protist correlations between many cytotrophs and their potential prey, in particular involving amoebae such as *Flamella* or unidentified variosean amoebas, some of which can form extensive cytoplasmic networks [65], and the cercozoans *Proleptomonas faecicola* or *Eocercomonas ramosa*. In parallel, many correlations linked ASVs belonging to the same genus, like the different unidentified *Amphifila*. While these could indicate possible trophic or ecological links between those ASVs, they could also be due to correlations between different ribotypes for the same protist genus. Although ASVs provide sorting of true biological sequences versus sequencing errors [66], it can lead to ribotypes of the same genus being considered as different ASVs and to assortativity, where nodes linked in a cluster belong to the same taxonomic group [50, 67]. Using a lower classification threshold for taxonomic units, such as grouping into 97% OTUs, could reduce the number of nodes and therefore edges, indicating clearer and biologically interpretable co-occurrences. Furthermore, the low taxonomic identification of many of the (protist) ASVs and the reduced, or simply absent, knowledge of the biology of many of the organisms represented in those networks doesn’t allow for further biological conclusions. In addition, functional assignments of soil protists still lag behind those of aquatic environments, making it difficult to ascertain trophic relationships [68].

In conclusion, this study attempts to identify differences in protist diversity and function between soils submitted to ambient and elevated atmospheric CO_2_ levels. Despite low replicate numbers and a reduced dataset, we showed that CO_2_ concentration modulates the (metabolically active) core consumer protist diversity in bulk and rhizosphere soils, but not particularly the function of groups identified therein. We also demonstrated that while we can’t conclude on the interactions between microorganisms under changing environmental conditions, co-occurrences are also closely related to CO_2_ conditions and that below-ground selection most likely results from aboveground impacts of atmospheric greenhouse gas concentrations and bottom-up effects of bacteria and resources’ availability. Future research would benefit from more back-to-the-basics study of (culturable) protists with microscopy combined with molecular studies, to develop and improve microeukaryotes’ functional assignments and better disentangle trophic interactions and subsequent eco-evolutionary dynamics in the context of global change. Given the complex interactions between carbon dioxide, plants, microorganisms, and soil processes, research is still needed to fully understand the consequences of elevated CO_2_ on soil environments.

## Supplementary Information

Below is the link to the electronic supplementary material.

Supplementary file1 (DOCX 4190 KB)

Supplementary file2 (CSV 9 KB)

## Data Availability

The dataset supporting this article's conclusions is included within the article and its additional files. Sequences used in this work can be found under the NCBI Project number PRJNA656997. Other data can be provided upon reasonable request.
