# Supplementary material for: Elevated Atmospheric Co2 Levels Impact Soil Protist Functional Core Community Compositions
Source: Curr Microbiol. 2024 Oct 16;81(12):411. doi: 10.1007/s00284-024-03930-3 (PMC11485191; doi:10.1007/s00284-024-03930-3)
Supplement: Supplementary file 1 — Supplementary file1 (DOCX 4190 KB) [file 284_2024_3930_MOESM1_ESM.docx]

ELEVATED ATMOSPHERIC CO_2_ LEVELS IMPACT SOIL PROTIST FUNCTIONAL CORE COMMUNITY COMPOSITIONS.

Alessandra Ö. C.-Dupont, David Rosado-Porto, Indhu Shanmuga Sundaram, Stefan Ratering, Sylvia Schnell

SUPPLEMENTARY INFORMATION

SI Table 1 – ASV table with the taxonomy of the core eukaryome (SI_Table1_ASVTax.csv)

Supplementary figures


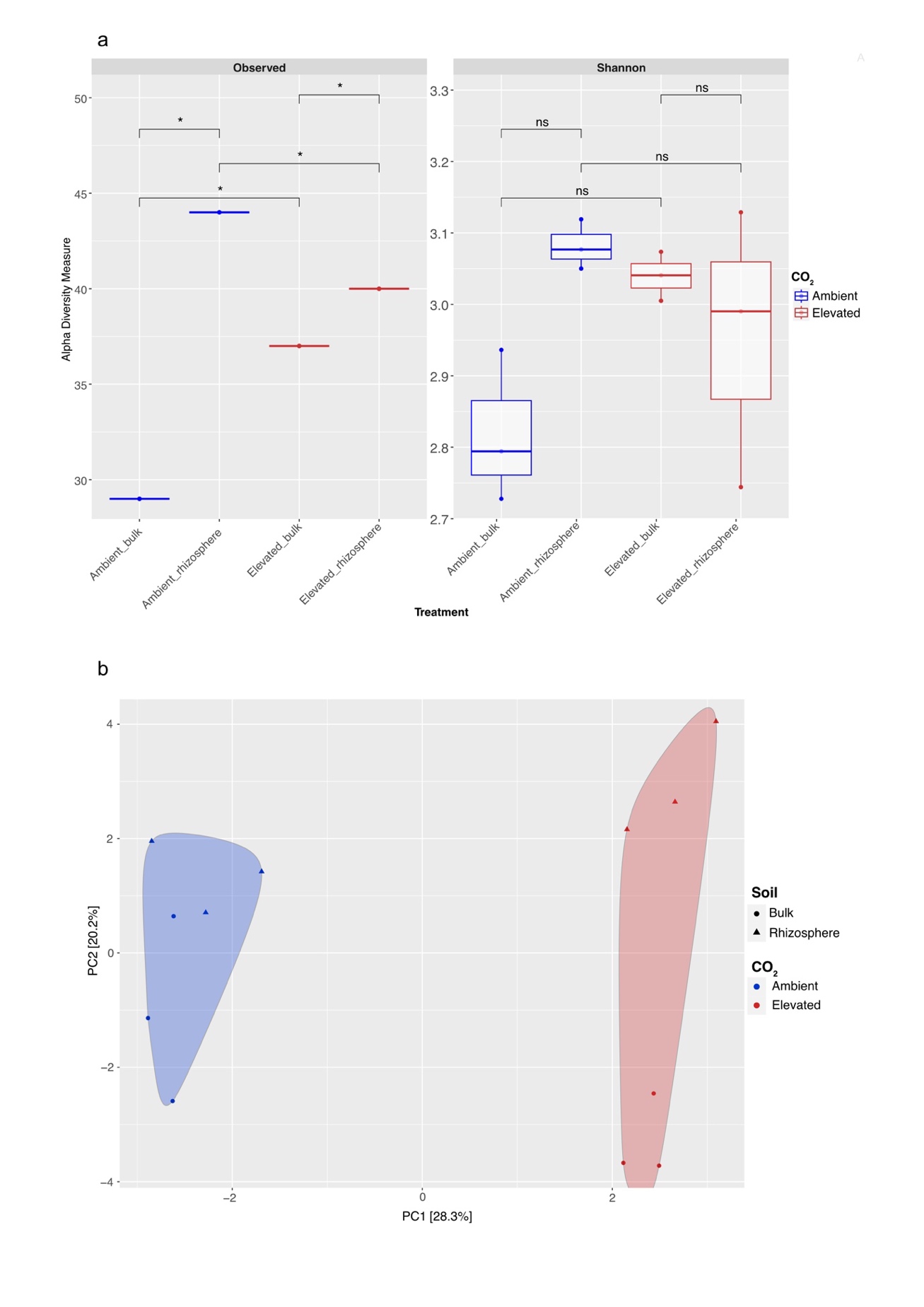


**SI Fig. 1** a) Alpha-diversity in rhizosphere and bulk soils under ambient (blue) and elevated (red) CO_2_ conditions. * : *p* < 0.01, ns: non-significant (*p* > 0.05); b) Beta-diversity principal component analysis for rhizosphere (triangles) and bulk (circles) soils under ambient (blue) and elevated (red) CO_2_ conditions.


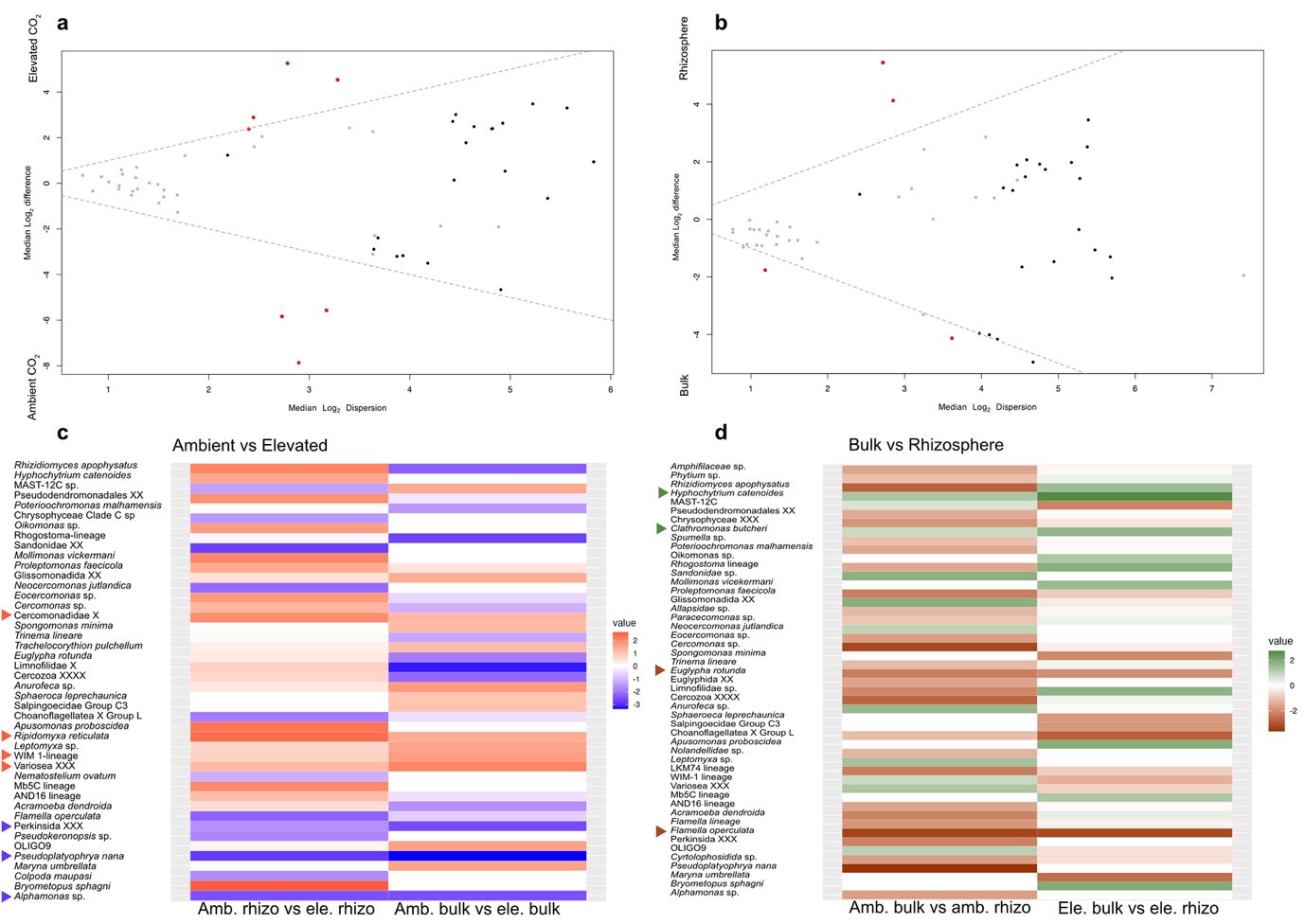


**SI Fig. 2** ALDEx2 differential abundances of core eukaryomes: MWplots for a) ambient vs elevated CO_2_ conditions and b) bulk vs rhizosphere soils. Red points indicate significantly differentially abundant ASVs, with an effect size >|2| and Bonferroni-corrected *p*-value < 0.1; black points indicate ASVs with an effect size>|2| but with a p-value > 0.1.

ALDEx2 differential abundances heatmaps for c) ambient vs elevated CO_2_ conditions in both rhizosphere (left column) and bulk (right column) soils and d) bulk vs rhizosphere soils under ambient (left column) and elevated (right column) CO_2_ conditions. For each column two conditions are compared; negative values indicate taxa enriched in the first condition of the comparison (i.e., in ambient rhizo vs elevated rhizo, negative values indicate that the taxon is enriched in samples from ambient CO_2_ conditions). Arrows indicate significantly differentially abundant taxa, corresponding to the red dots in MW plots (red: enriched under elevated CO_2_ conditions, blue: enriched under ambient CO_2_ conditions, green: enriched in rhizosphere soils, brown: enriched in bulk soils).


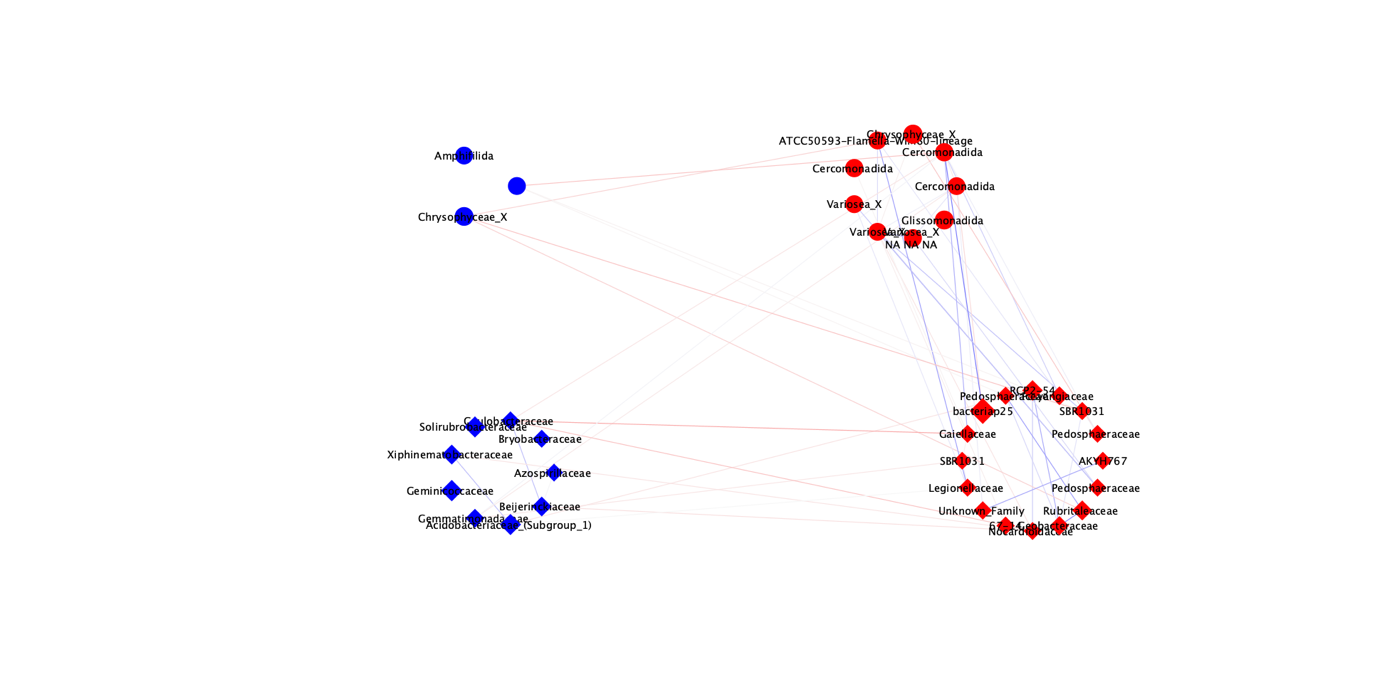


**SI Fig 3** “Hub” nodes co-occurrence network between protists (circles) and bacteria (diamonds) at elevated (red) and ambient (blue) CO_2_ conditions: only nodes showing a degree of >10 in the main co-occurrence network (Fig 6a) are shown. Blue edges represent positive co-occurrences, and red edges represent negative co-occurrences.


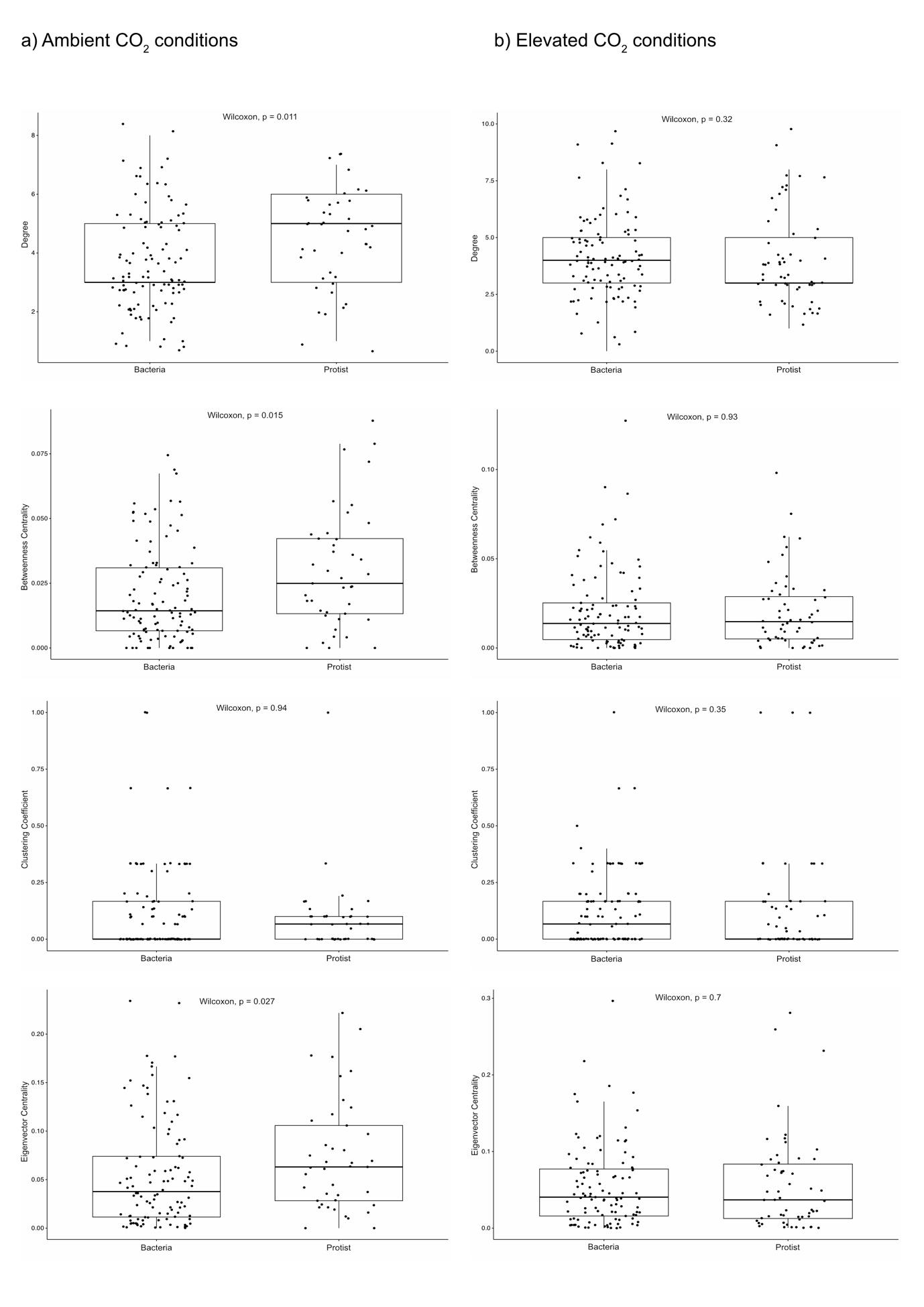


**SI Fig 4** Comparison of network topological features between protist and bacteria under a) ambient CO_2_ conditions and b) elevated CO_2_ conditions.


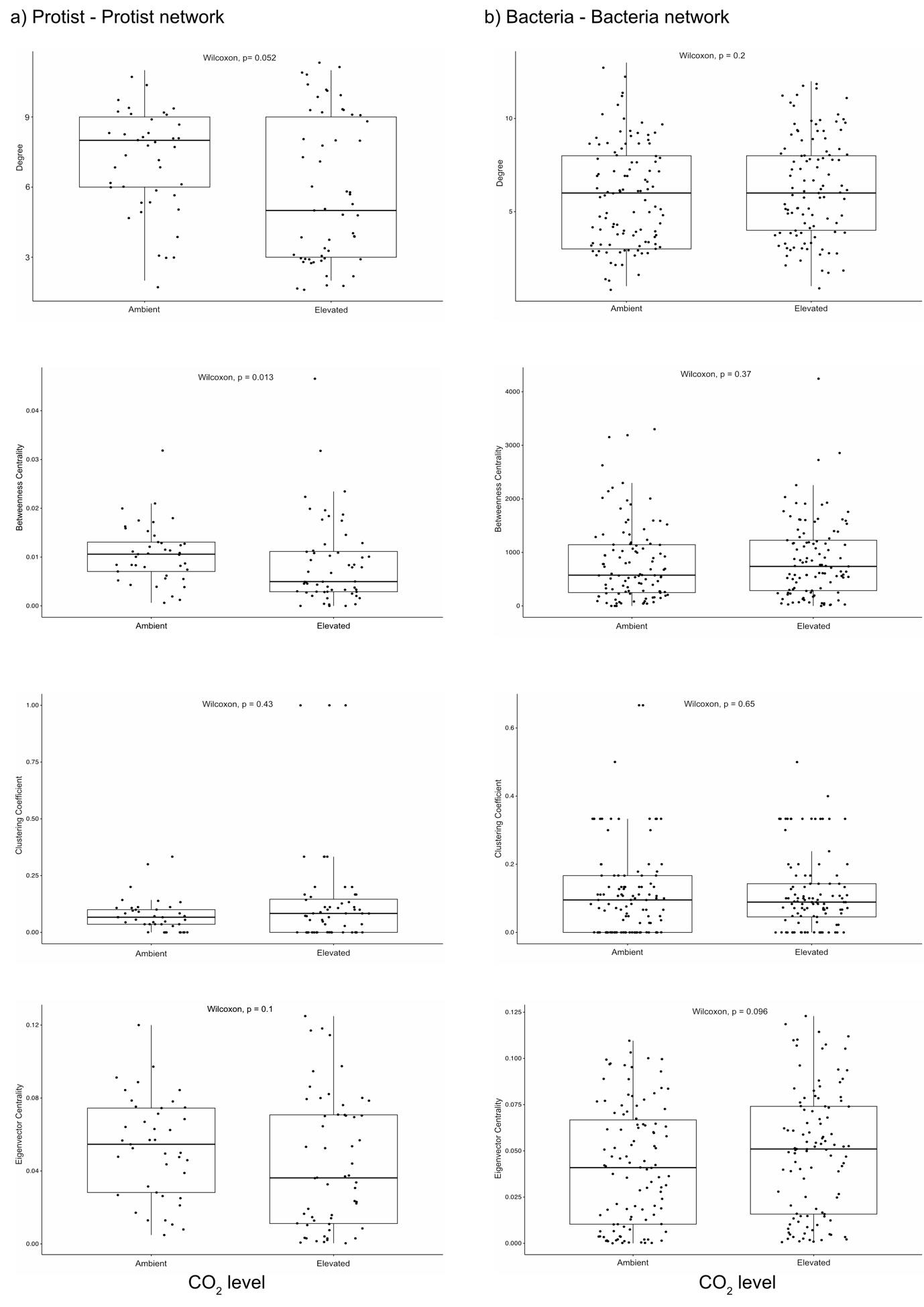


**SI Fig. 5** Comparison of network topological parameters under ambient and elevated CO2 conditions for a) protist-protist and b) bacteria–bacteria networks


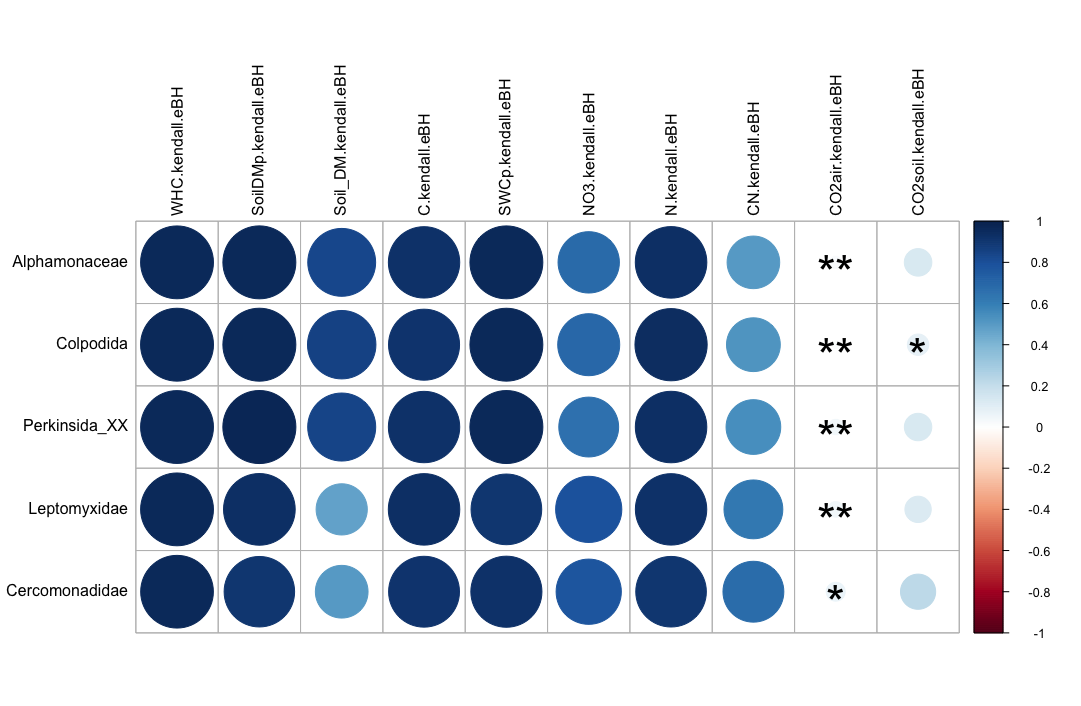


**SI Fig 6** Kendall correlation of significantly differentially abundant (ALDEx2) ASVs with environmental parameters. WHC: water holding capacity (g H_2_O g^-1^ soil), SoilDM: soil dry matter (g), SoilDMp: soil dry matter (%), C: total carbon (%), SWCp: soil water level (% of WHC), NO3: NO_3_^-^ content (µmol g_DW_^-1^), N: total nitrogen (%), CN: carbon nitrogen ratio, CO2air: CO_2_ air concentration (ppmV), CO2soil: CO_2_ soil flux (µmol m^-2^ s^-1^ec)
